# Development and Evaluation of a Virtual Reality Program for Immediate Newborn Care Training in Nursing Education: A Feasibility Study

**DOI:** 10.5334/pme.1538

**Published:** 2024-12-10

**Authors:** Hsiao-Ying Hung, Yu-Wen Wang, Min-Chai Hsieh, Po-Yu Chen, Ying-Ju Chang

**Affiliations:** 1 Department of Nursing, College of Medicine, National Cheng Kung University, Tainan, Taiwan; 2 Department of Nursing, National Cheng Kung University Hospital, Tainan, Taiwan; 3 Department of Nursing, College of Health Sciences, Chang Jung Christian University, Tainan, Taiwan; 4 The Department of Multimedia and Animation, Tainan University of Technology, Taiwan; 5 The Department of Nursing, Min-Hwei College of Health Care Management, Tainan, Taiwan; 6 Institute of Allied Health Sciences & Department of Nursing, College of Medicine, National Cheng Kung University, Tainan, Taiwan

## Abstract

**Introduction:**

Nursing education equips students with the skills necessary to navigate the clinical environment. Repetitive training in complex skills ensures patient and student safety. Immersive virtual reality (VR) offers a realistic and safe environment for such repetitive learning processes. However, the feasibility of integrating such technology into the training of immediate newborn care skills remains unexplored.

**Methods:**

In this feasibility study, the care procedure for immediate newborn care (INC) was standardized and converted into a VR teaching model. Experts and students were then recruited to assess and evaluate the suitability, usefulness and user-friendliness of the INC-VR model. A total of 35 students were recruited and allocated to a VR learning group and a traditional learning group to evaluate the INC-VR model in terms of knowledge acquisition, skill confidence, performance accuracy, and the time required to complete the INC tasks.

**Results:**

Thirteen INC care tasks were transformed into a 15-minute INC-VR model, and the suitability, usefulness, and user-friendliness of the model were validated by both students and experts. Furthermore, students in the VR group demonstrated comparable INC knowledge, confidence, and performance accuracy to those in the traditional group, with a more time-efficient learning framework (10.3 minutes vs. 35 minutes).

**Discussion:**

The INC-VR model developed herein can supplement traditional teaching to enhance students’ learning. This model could provide an accessible platform for additional practice and remediation, addressing the limitations of real-time skill practice opportunities. Therefore, it may also serve as a valuable reference for other institutions developing similar VR educational tools.

## Introduction

The goal of nursing education is to equip future nurses with practical competencies [1]. However, the dynamic and unpredictable nature of health care delivery presents significant challenges to clinical teaching, thus impeding systematic learning and leading to disparate experiences among students. To address these challenges, simulation-based education (SBE) has emerged as a crucial approach in nursing education. SBE allows educators to systematically design and replicate vital scenarios to enhance students’ ability to handle real-world situations and offers a safe environment for students to reinforce learning [2,3]. The educational benefits of SBE have been supported by experiential learning theory [4] and several studies [5,6]. However, each repetition of a face-to-face SBE requires intensive resources, including time, equipment, and space [7], which could impede the feasibility of SBE education.

With technological advances and the increased affordability of related devices, virtual reality (VR) has been gradually applied in healthcare education [8,9]. Virtual simulation provides students with an accessible and repeatable learning environment to develop specific competencies through the integration of computer technologies [10]. Research has shown that virtual simulation can enhance nursing students’ knowledge, psychomotor skills, psychosocial abilities, and self-efficacy [11,12]. Despite these benefits, the definition of VR in education varies widely. According to the Healthcare Simulation Standards of Best Practice™, VR is a computer-generated environment that allows learners to experience various stimuli through specialized equipment [13]. However, existing reviews have used various definitions of VR, including computer-based and web-based programs, across different student populations. This lack of consistency may contribute to uncertainty about the effectiveness of VR in nursing education. Additionally, the potential impact of VR on midwifery and maternal care education is further restricted by the absence of sufficient research on the topic [8,9].

Immediate newborn care is critical for the survival and development of infants [14]. However, newborn care involves complex and critical tasks, such as tactile stimulation, temperature maintenance, mucus suctioning, Apgar score assessment, and umbilical cord care, thus making teaching difficult within the limited time available while also further restricting students’ learning opportunities. In countries with low birth rates, such as Taiwan, the unique reproductive context and scarcity of hands-on experience exacerbate the difficulties in mastering these specific abilities. Limited learning opportunities can lead to student frustration during clinical internships, a critical element that affects students’ professional commitment to nursing and further influences their career decision-making in the nursing field [15,16]. To address these gaps, we aimed to develop an immediate newborn care VR (INC-VR) model to complement our teaching and tested its usability and feasibility in undergraduate nursing education.

## Methods

### Design

A three-phase feasibility study was conducted from August 2020 to October 2022 at a university in southern Taiwan, following ethics committee approval (NCKU HREC-E-109–085-2). In phase 1, the care procedure for immediate newborn care (INC) was standardized and converted into a VR teaching model. In phase 2, maternal experts and undergraduate nursing students were recruited to evaluate the suitability, usefulness, and user-friendliness of the INC-VR model. In phase 3, thirty-five students were recruited and allocated to a VR learning group and a traditional learning group to evaluate the feasibility of the INC-VR model.

### Phase 1: Development of the INC-VR model

According to a synthesis of information from references, textbooks, and care guidelines, the immediate newborn care procedures were standardized. The care procedures include 13 core task principles with 50 skill demonstrations (Table 1). Six maternal nursing experts with more than ten years of experience in nursing education and clinical practice were subsequently invited to assess the suitability of the INC procedure via the content validity index (CVI) [17]. Overall, the CVI for each skill demonstration ranged from 0.8 to 1.0.

**Table 1:** Standardization of core nursing tasks for immediate newborn care.


TASKS SEQUENCE	CORE TASK PRINCIPLES	STEPS, N	NECESSARY EQUIPMENT AND SUPPLIES

**Before Newborn Delivery**	Preparing equipment and supplies	15	Warmer, heat lamps, sterile drapes, surgical cap, surgical mask, sterile gown, aspirator, umbilical clip, umbilical scissors, cotton swab, gauze, sterile gloves, 75% alcohol, Vaseline, a rectal thermometer, a warming cap and wristbands

	Compliance with sterile principles		

	Maintaining a Proper Warm Environment		

**After Newborn Delivery**	Safely hold newborn	2	

	Maintain continuous warmth	1	

	Maintain clear airways	2	

	Assess Apgar Score correctly	5	

	Perform umbilical cord care correctly	5	

	Temperature measurements	2	

	Conduct a thorough physical examination	8	

**Newborn to mother**	Accurate identity verification	4	

	Facilitate skin-to-skin contact	2	

	Complete newborn record sheet correctly	4	


After seven key steps of the INC procedure were clarified according to the experts’ suggestions, the INC-VR model was developed in collaboration with information technology experts. The INC-VR model was created with Unity, the OpenVR development kit, and the SteamVR driver for rendering VR graphics. The Unity XR Interaction Toolkit was utilized to generate interactive objects, and the Universal Render Pipeline enhanced lighting, shadows, and material rendering. Given that the INC-VR model was designed to enable instructors to effectively demonstrate immediate newborn care procedures during the course, as well as to facilitate students’ independent learning, it included a comprehensive checklist outlining the INC steps, accompanied by detailed textual explanations and a color-coded reminder system. Finally, a 15-minute INC-VR model with thirteen INC care tasks was developed (Appendix 1).

### Phase 2: Evaluating the suitability, usefulness, and user friendliness of the INC-VR model

Six experts with experience in teaching immediate newborn care and twenty-eight nursing students trained in immediate newborn care during 2021 participated in the evaluation. To assess the model’s suitability, we first developed and administered the INC-VR suitability assessment questions, guided by the educational theory of Understanding by Design (Wiggins & McTighe, 2011) [18], an outcome-oriented approach that employs backward design to align instructional activities and assessments with learning objectives. Additionally, we adopted Davis’ (1989) [19] questionnaire to measure the perceived usefulness and ease of use of the INC-VR.

### Measurements and study procedure

The experts were invited to operate the INC-VR model and then assess its suitability through five questions developed by the researchers: (1) I believe that the INC-VR model’s design achieves the desired learning outcomes; (2) I believe that the INC-VR model’s content effectively achieves the intended educational objectives; (3) I believe that the interactive design of the INC-VR model enhances students’ learning; (4) I believe that the evaluation design of the INC-VR model reflects the students’ learning outcomes; and (5) Overall, I believe that the INC-VR model helps students master immediate newborn care skills. Each item was assessed via a 5-point Likert scale ranging from 1 (strongly disagree) to 5 (strongly agree), with a higher mean score indicating greater suitability of the INC-VR model.

After the INC-VR model was modified based on experts’ suggestions, 28 students with prior experience in immediate newborn care were recruited to operate the INC-VR model and to assess its usefulness and user-friendliness. The assessment utilized modified questions from Davis’ (1989) questionnaire on perceived usefulness and ease of use [19]. For usefulness, the students rated to the following items: (1) The INC-VR model helped me master the key learning points more easily; (2) The INC-VR model increased my proficiency in immediate newborn care; (3) The INC-VR model made my learning process smoother; (4) The INC-VR model improved my confidence in performing this skill; and (5) Overall, INC-VR model was useful to me. Each item was assessed on a 5-point Likert scale ranging from 1 (strongly disagree) to 5 (strongly agree), with higher scores indicating that students perceived the INC-VR as useful. The Cronbach’s α for usefulness scale in this study was 0.80. For ease of use, the students responded to the following items: (1) The functions provided by the INC-VR model are easy to use; (2) The operation interface of the INC-VR model is simple and easy to understand; (3) I quickly learned how to use the INC-VR model; (4) I encountered no difficulty in operating the INC-VR model; and (5) Overall, the INC-VR model is easy to use. Each item was also assessed on a 5-point Likert scale ranging from 1 (strongly disagree) to 5 (strongly agree), with higher scores indicating that the students found the VR model easy to use. The Cronbach’s α for ease of use scale in this study was 0.95.

### Results for the suitability, usefulness, and user-friendliness of the INR-VR model

As Table 2 shows, the experts’ mean scores for the suitability of the INC-VR model ranged from 4.0 to 4.3, indicating that the experts agreed that the content and design of the INC-VR model were appropriate and capable of enhancing student learning. Furthermore, the students’ mean score for usefulness and user-friendliness of the INC-VR model ranged from 3.8 to 4.3 and 4.4 to 4.6, respectively, indicating that the students perceived the INC-VR model as a user-friendly system that could facilitate their learning.

**Table 2:** Suitability, Usefulness and user-friendliness Evaluation of the INC-VR.


ITEMS	SCORE

**Suitability (n = 6)**	

1. I believe that the INC-VR model’s design achieves the desired learning outcomes.	4.3 ± 0.5

2. I believe that the INC-VR model’s content effectively achieves the intended educational objectives.	4.3 ± 0.5

3. I believe that the interactive design of the INC-VR model enhances students’ learning.	4.0 ± 0.9

4. I believe that the evaluation design of the INC-VR model reflects the students’ learning outcomes.	4.2 ± 0.8

5. Overall, I believe that the INC-VR model helps students master immediate newborn care skills.	4.2 ± 0.4

**Usefulness (n = 28)**	

1. The INC-VR model helped me master the key learning points more easily.	4.1 ± 0.9

2. The INC-VR model increased my proficiency in immediate newborn care.	4.0 ± 0.7

3. The INC-VR model made my learning process smoother.	4.2 ± 0.7

4. The INC-VR model improved my confidence in performing this skill.	3.8 ± 1.0

5. Overall, INC-VR model was useful to me.	4.3 ± 0.7

**User-friendliness (n = 28)**	

1. The functions provided by the INC-VR model are easy to use.	4.4 ± 0.7

2. The operation interface of the INC-VR model is simple and easy to understand.	4.5 ± 0.6

3. I quickly learned how to use the INC-VR model.	4.5 ± 0.6

4. I encountered no difficulty in operating the INC-VR model.	4.4 ± 0.7

5. Overall, the INC-VR model is easy to use.	4.6 ± 0.6


*Note:* VR = virtual reality, INC = Immediate Newborn Care, each item was assessed using a 5-point Likert scale ranging from 1 (strongly disagree) to 5 (strongly agree); Data was shown as mean ± standard deviation.

### Phase 3: Evaluating the feasibility of the INC-VR model

After optimizing the INC-VR model based on feedback from experts and students, 35 undergraduate nursing students aged ≥ 20 years, with no prior experience in immediate newborn care and enrolled in the required obstetric nursing course, were recruited to evaluate the model’s feasibility in terms of its impact on knowledge, skill confidence, and performance. Students with a history of VR-related side effects or visual or auditory impairments were excluded. To ensure alignment with learning objectives, we developed the INC knowledge scale, and the INC care procedures checklist based on the educational theory of Understanding by Design [18] to assess students’ knowledge and competency in immediate newborn care. Additionally, Grundy’s (1993) Skill Confidence Scale [20] was modified to assess students’ confidence in performing INC skills. To eliminate study bias, a traditional learning group was recruited before the development of the INC-VR model, and the VR group recruited the following year.

### Measurements and study procedure

A set of questionnaires comprising four subscales was used to collect data on participants’ demographics, INC knowledge, skill confidence, and performance. The characteristics of the students, including age, gender, previous clinical experience with newborns, experience with digital learning systems, and prior experience with virtual reality, were collected.

The INC knowledge scale consists of ten multiple-choice questions assessing students’ knowledge of key components of the Apgar score (timing, heart rate, breathing rate, muscle tone, reflexes, and skin color) and the principles of umbilical cord care. Each correct answer was awarded one point, with higher scores indicating greater INC knowledge. The Skill Confidence Scale, which consists of five questions modified from Grundy’s (1993) [20] scale, was used to assess students’ confidence in performing INC skills. The five questions were the following: (1) I am confident that my skill performance is accurate; (2) I perform this skill without hesitation; (3) My performance demonstrates my skill proficiency to others; (4) I am confident while performing this skill; and (5) I am satisfied with my performance. Each item is rated on a 5-point Likert scale ranging from 1 (no confidence) to 5 (very confident), with total scores ranging from 5 to 25. Higher scores indicate greater confidence in INC skills. Students’ skill performance was evaluated by assessors, who independently rated the accuracy of their INC skills based on recorded videos of their skill tests. The evaluation utilized a standardized INC care procedures checklist (Table 1). The time taken to complete each skill was also recorded.

The validity of the above questionnaire was evaluated by five maternal education experts using the content validity index (CVI), with scores ranging from 0.8 to 0.9. The Cronbach’s α for Skill Confidence Scale in this study was 0.84. For students’ skill performance, the interrater reliability coefficient (Cohen’s kappa) was 0.80.

**Figure 1:**
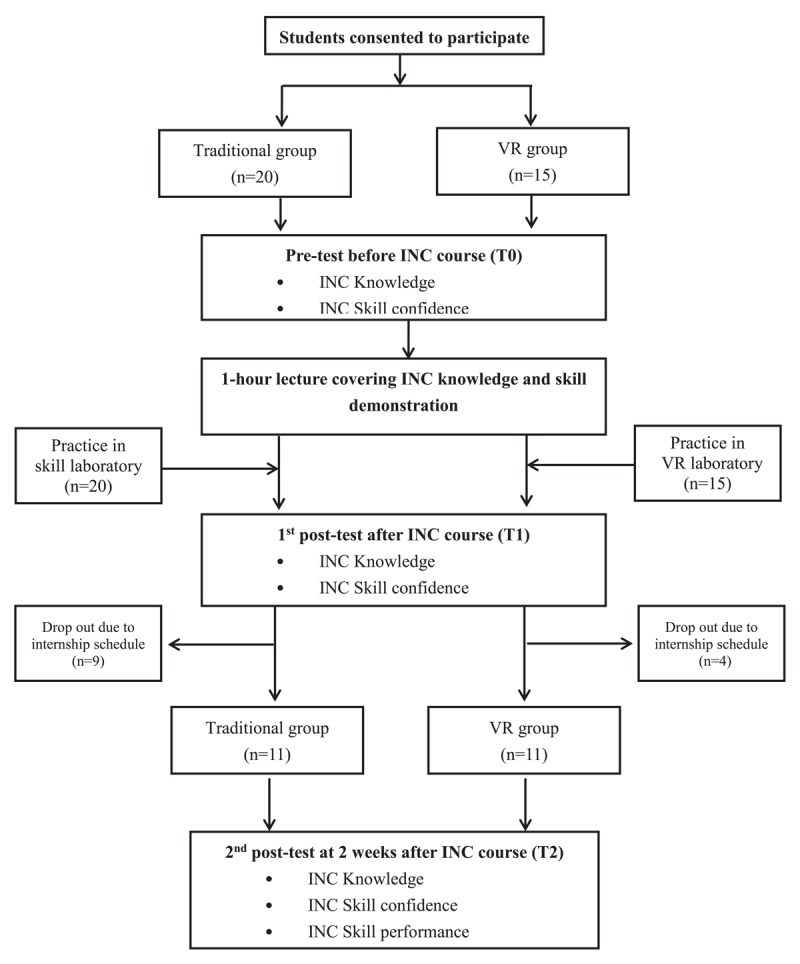
The procedure of 3rd phase feasibility study.

Figure 1 illustrates the study process for testing the feasibility of the INC-VR model.

Before the lecture, all students completed the INC knowledge and Skills Confidence Scale as a pretest (T0). Following the pretest, both groups attended a one-hour lecture covering INC knowledge and a demonstration of the required skills. Afterward, students in each group participated in a three-hour skill training session, in which each student was required to complete at least one full practice of the INC skill procedure. Due to equipment limitations, a maximum of two students could practice simultaneously, allowing for structured rotation during the session. Students in the traditional group practiced immediate newborn care with a newborn model and relevant newborn care equipment in a nursing skills laboratory, with the assistance of two teaching assistants who were available to answer their questions. Students in the VR group received a 20-minute tutorial on how to use the VR system and then practiced immediate newborn care using the INC-VR program with HTC headsets and hand controllers. Two teaching assistants were available in the VR classroom to assist with technical issues. On average, each student in the VR group spent 10.3 minutes completing the skill practice, and students in the traditional group spent an average of 35 minutes. After the skill practice sessions, both groups completed the INC knowledge scale and the INC Skills Confidence Scale as their first posttests (T1). Two weeks later, both groups repeated the posttests as their second posttests and participated in a demonstration to assess their skill performance (T2).

### Results for the feasibility of the INC-VR model

Twenty students participated in the traditional teaching group in 2021, and 15 participated in the VR group in 2022. The average age was 20.4 ± 1.6 years, with the majority being female (82.9%, n = 29). Nearly 70% (n = 25) had experience with digital learning platforms such as Moodle and WebEx. Over one-third (n = 12) had previously used VR, primarily for entertainment, while 25% (n = 9) had used VR for educational purposes. The Wilcoxon rank sum test revealed no significant differences in characteristics between the two groups (Appendix 2).

All the students completed the pretest (T0) and the first posttest (T1). However, by the time of the second data collection (T2), some students had already started their obstetrics internships and, thus, may have gained additional clinical experience and knowledge. To mitigate potential bias from these clinical experiences, only 11 students in the traditional group (45% dropout rate) and 11 students in the VR group (26.7% dropout rate) finished the second posttest and skill assessments (Figure 1). There were no significant differences in age, gender, digital learning experience, or prior VR use between those who dropped out and those who completed the study.

The Wilcoxon rank sum test was used to compare the differences between the two groups in terms of INC knowledge, skill confidence, skill accuracy, and skill execution time. No statistically significant differences were found between the groups across all measured outcomes, indicating that students in the VR group performed comparably to those in the traditional teaching group (Appendix 3).

The Friedman rank-sum test was applied to assess changes in knowledge and confidence within both groups across the three tests, and post hoc pairwise comparisons within each group were conducted using the pairwise Wilcoxon signed-rank test with Bonferroni correction. As illustrated in Appendix 4, INC knowledge in the VR group significantly increased from 5.4 (SD = 1.6) at T0 to 8.9 (SD = 0.8) at T1 (*p* < 0.001) and then remained stable at 9.3 at T2. Similarly, confidence scores significantly improved from 13.5 at T0 to 17.9 at T1 (*p* < 0.001) but then notably decreased to 12.2 at T2. In the traditional group, INC knowledge also significantly increased from 5.7 (SD = 1.7) at T0 to 9.0 (SD = 0.6) at T1, remaining stable at 9.2 at T2. However, confidence levels did not significantly change across the three assessments. These results suggest that while VR training effectively enhances initial confidence and knowledge, sustained confidence may require additional reinforcement.

## Discussion

A 15-minute INC-VR model comprising 13 core tasks for immediate newborn care was developed. The model’s suitability, usefulness, and user-friendliness were validated by maternal care experts and undergraduate nursing students. Additionally, the feasibility of using the INC-VR model to support students’ post-class skill practice was preliminarily confirmed, as students who engaged with the INC-VR model demonstrated comparable levels of INC knowledge, skill confidence, and performance, all within a more time-efficient learning framework. Therefore, we believe that our INC-VR model can serve as a supportive and accessible teaching material for undergraduate nursing students learning immediate newborn care skills.

The benefits of our VR model for learning stem from a development process that adhered to standardized INC procedures, incorporated user feedback, and was guided by the principles of the Technology Acceptance Model (TAM) [21]. As a result, our VR system’s interface and operation were designed to be straightforward, with clear user guides provided to reduce technical barriers for users. This simplification could help mitigate the challenges often faced when integrating innovative instructional materials into nursing education [22], making the INC-VR model more accessible for undergraduate nursing students. Meanwhile, the INC-VR model offers a highly immersive environment that enhances student concentration and learning through multisensory stimulation, as supported by multimedia learning and immersion theories. Its interactive design provides real-time feedback, allowing students to actively build knowledge through exploration and interaction [23,24]. These features make the learning outcomes achieved with the INC-VR model comparable to those of traditional practice.

While the INC-VR model demonstrates notable benefits, several limitations should be considered. First, our preliminary findings were based on a relatively small sample size and a single practice session, designed to minimize potential influence from students’ clinical internship experiences on the study results. Given the importance of repeated exposure in skill-based learning, future applications of the INC-VR model should focus on creating opportunities and incorporating game-based learning to encourage students to engage in self-directed, repetitive practice. Additionally, breaking down the complexity of INC procedures into distinct tasks may allow students to gradually master their skills [25]. This approach could optimize information presentation and minimize excessive cognitive load, while also addressing the varied training requirements of different nursing schools. Furthermore, the lack of haptic feedback in the INC-VR model may reduce immersion levels, potentially resulting in fewer natural interactions by users. To enhance the learning experience, future developments could consider incorporating a haptic feedback module, allowing users to engage in more realistic and immersive interactions that better simulate hands-on skills [26,27]. This study represents an initial feasibility trial, and further formal research is needed to rigorously confirm the educational effectiveness of the INC-VR program and explore any undesirable effects, such as cybersickness symptoms.

## Conclusion

This study confirmed the feasibility of a 15-minute INC-VR model comprising 13 core tasks as a supplementary tool to traditional teaching in immediate newborn care. By offering an accessible platform for additional practice and remediation, the VR model could address the limited real-time opportunities for skill practice, empowering students to independently reinforce and refine their skills. Therefore, the INC-VR model may serve as a valuable reference for other institutions developing similar VR educational tools. While promising, further rigorous research is needed to confirm the educational effectiveness of the INC-VR model and to investigate potential negative effects, such as cybersickness.

## Additional Files

The additional files for this article can be found as follows:

10.5334/pme.1538.s1 Supplementary File 1. Appendix 1–3.

10.5334/pme.1538.s2 Supplementary File 2. Appendix 4.
