# Supplementary material for: Development and Evaluation of a Virtual Reality Program for Immediate Newborn Care Training in Nursing Education: A Feasibility Study
Source: Perspect Med Educ. 2024 Dec 10;13(1):620–8. doi: 10.5334/pme.1538 (PMC11639688; doi:10.5334/pme.1538)
Supplement: Supplementary File 1. — Appendix 1–3. [file pme-13-1-1538-s1.pdf]

Appendix 1. Examples of Screen Shots from Immediate Neonatal Care VR

An INC training task in a Spontaneous Vaginal Delivery Scenario

Miss Wang, G1P0, is experiencing regular contractions. Her cervix is now fully dilated to 10 cm, and the fetal station is +3. She is being taken to the delivery room for a vaginal birth.

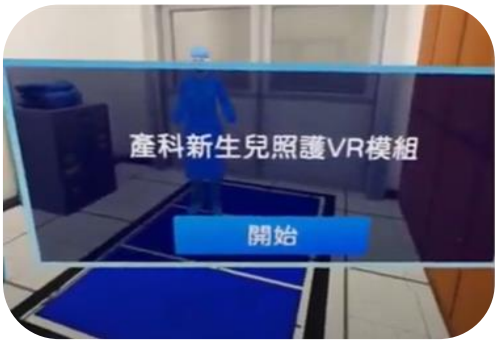

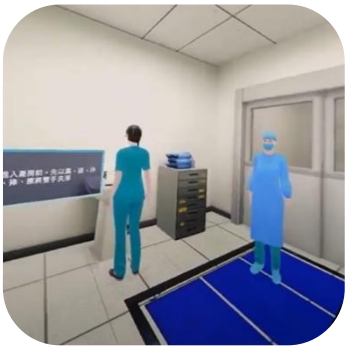

Preparation before Newborn Delivery

- **Preparation necessary equipment and supplies**

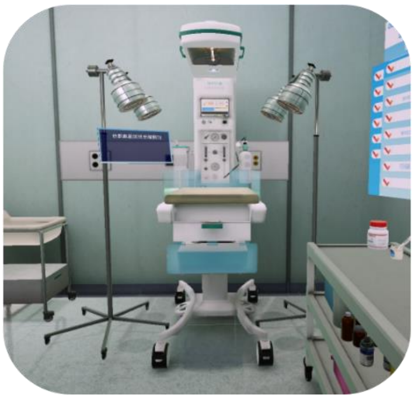

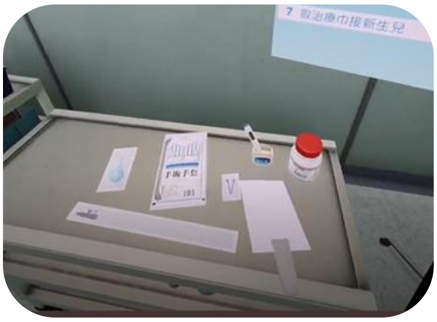

- **Dry the infant and demonstrate mucous suction.**

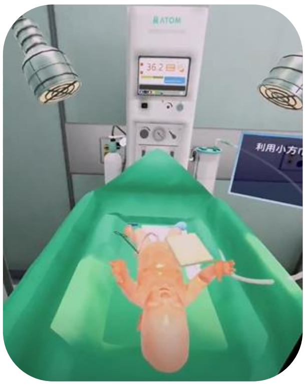

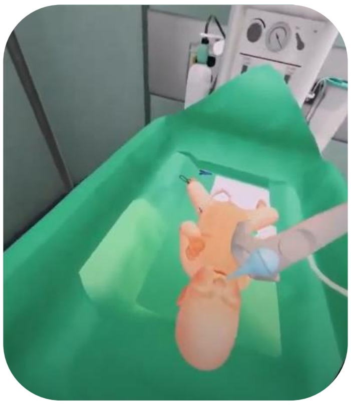

- 1st & 5th minute Apgar Score assessment.

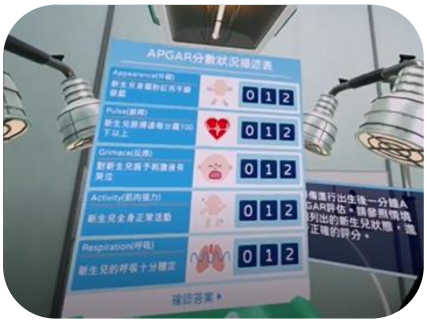

- Facilitate skin-to-skin contact

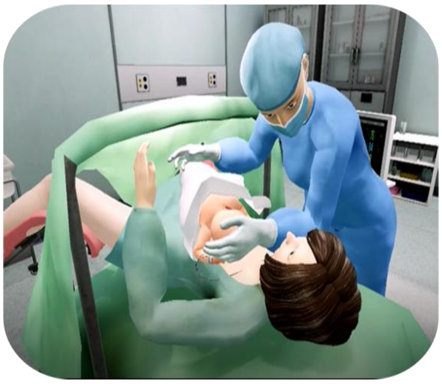

- Guiding checklist and color for INC process.

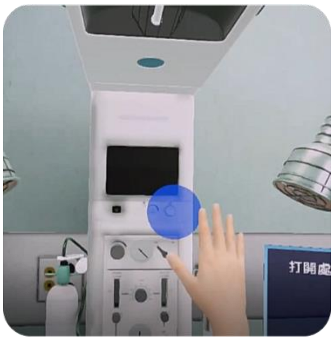

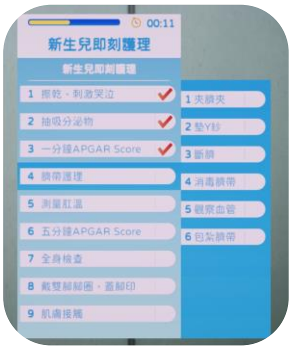

Appendix 2. Demographic characteristics of the students in Phase 2 study (n = 35)

Variable	Total (n= 35)	INC-VR group (*n* = 15)	Control group (*n* = 20)	*p*
Age (Mean ± SD)	20.4 ± 1.6	21.0 ± 2.3	20.0 ± 0.3	.009 [1]
Gender				.999 [2]
Female (*n*, %)	29 (82.9)	12 (80)	17 (85)	
Digital platform learning experience				.144 [2]
Yes	24 (68.6)	8 (53.3)	16 (80)	
VR experience				.282 [2]
Yes	12 (34.3)	7 (46.7)	5 (25)	

Note: [1] Wilcoxon rank sum test, [2] Fisher's exact test. SD=standard deviation

Appendix 3. Comparison of INC knowledge, skill confidence and skill performance between the VR and control group (n = 35)

	VR group (*n* = 15)	Control group (*n* = 20)	p^1
INC Knowledge (0-10)			
Pretest (T0)	5.5 ± 1.5	5.6 ± 1.6	.811
1st Posttest (T1)	8.8 ± 0.9	9.0 ± 0.7	.359
2nd Posttest (T2)[2]	9.3 ± 0.6	9. 2 ± 0.8	.828
INC Skill Confidence (0-25)			
Pretest (T0)	13.1 ± 3.7	11.4 ± 5.0	.353
1st Posttest (T1)	17.1 ± 3.5	15.7 ± 2.7	.173
2nd Posttest (T2)[2]	12.2 ± 5.7	13.4 ± 4.9	.276
INC Performance[2]			
Accuracy (0-25)	15.2 ± 3.2	15.7 ± 4.5	.479
Execution time (Seconds)	1061 ± 125	1011 ± 168	.721

Note: [1] Wilcoxon rank sum test; [2] Only 11 students completed the second posttest and the skill performance test in both the VR and traditional groups; Data was shown as mean ± standard deviation; IVR=Immersive virtual reality; INC=Immediate Newborn Care.
