# Supplementary figures and images for: Development and Evaluation of a Virtual Reality Program for Immediate Newborn Care Training in Nursing Education: A Feasibility Study
Source: Perspect Med Educ. 2024 Dec 10;13(1):620–8. doi: 10.5334/pme.1538 (PMC11639688; doi:10.5334/pme.1538)

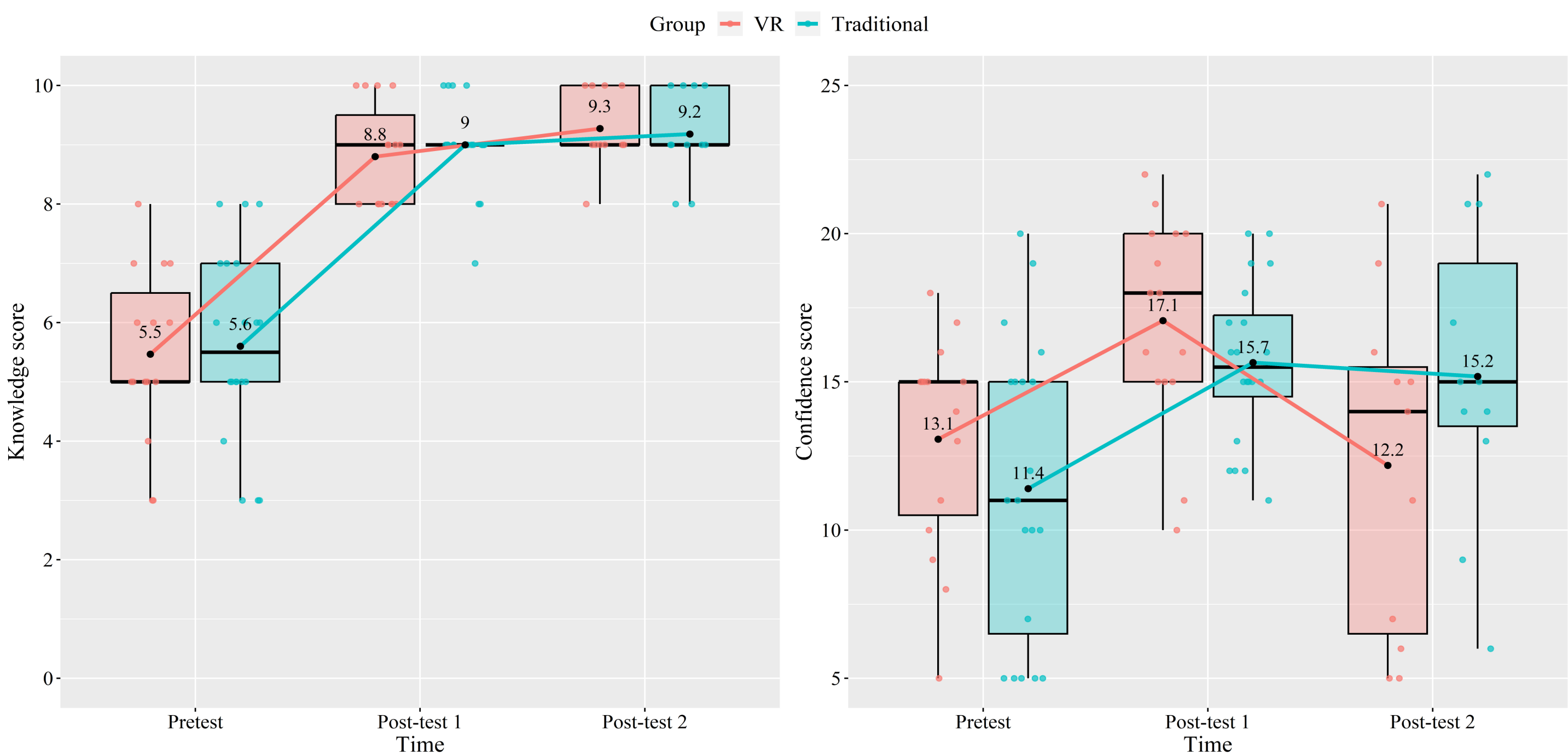

Figure 3. Change trajectory of immediate newborn care knowledge and confidence by groups.

Supplement: Supplementary File 2. — Appendix 4. [file pme-13-1-1538-s2.pdf]
